# From fear and vulnerability to fortitude: sustaining psychological well-being in the face of the COVID-19 pandemic

**DOI:** 10.1177/00812463221137876

**Published:** 2022-11-27

**Authors:** Anita Padmanabhanunni, Tyrone B Pretorius

**Affiliations:** Department of Psychology, University of the Western Cape, Republic of South Africa

**Keywords:** Fear of COVID-19, fortitude, perceived vulnerability to disease, psychological distress

## Abstract

Despite the societal increase in mental health disorders during the initial stages of the COVID-19 pandemic, many individuals were able to cope effectively with new mental health challenges. The heterogeneity in responses to adversity underscores the influence of protective factors in promoting coping behaviour. The current study investigates fortitude as a potential protective resource by examining the potential direct, mediating, and moderating roles of fortitude in the relationship between perceived vulnerability to disease, fear of COVID-19, and indices of psychological well-being. Participants (*n* = 355) were schoolteachers who completed the Perceived Vulnerability to Disease Questionnaire, Fear of COVID-19 Scale, Fortitude Questionnaire, UCLA Loneliness Scale, trait scale of the State-Trait Anxiety Inventory, Beck Hopelessness Scale, and the Center for Epidemiological Studies Depression Scale. Path analysis indicated that fortitude had a health-sustaining effect that was evident in its association with all indices of psychological well-being. Fortitude also mediated the relationship between perceived vulnerability to disease and depression, anxiety, and loneliness. In addition, fortitude moderated the relationship between fear of COVID-19 and depression. The significant moderating and mediating effects of fortitude on psychological outcomes support its potential for counterbalancing the negative mental health impacts of COVID-19. Interventions aimed at enhancing fortigenic appraisals of self and others may prove beneficial in promoting psychological well-being.

A significant body of research has emerged regarding the mental health impact of the COVID-19 pandemic (e.g., Zhang & Chen, 2021). Studies among various population groups and in a range of countries (Chen et al., 2021; Pretorius & Padmanabhanunni, 2021b) have confirmed that the pandemic has precipitated elevated levels of psychological distress in the form of heightened anxiety, depression, loneliness, and post-traumatic stress disorder (PTSD). For example, longitudinal studies of the general population in the United Kingdom (O’Connor et al., 2021; Pierce et al., 2020) confirmed that mental health deteriorated in the initial stages of the pandemic, characterized by elevated levels of anxiety, depression, and suicidal ideation. Similarly, cross-sectional studies of adults in the United States (Vahratian et al., 2021), China, and Spain (Wang et al., 2021) have reported heightened levels of anxiety and depression. In addition, problematic coping strategies, especially increased alcohol use, have been identified in several studies (Capasso et al., 2021). Mental health disorders have also been associated with an increased risk of COVID-19-related mortality (Fond et al., 2021).

Study findings (e.g., Pretorius & Padmanabhanunni, 2021b) in developing countries in Africa suggest that psychological distress is higher in these settings than in other areas of the world. A systematic review of mental health disorder prevalence rates during the pandemic in key regions in Africa, South Asia, and Latin America (Zhang & Chen, 2021) reported comparatively high rates of mental health disorders, particularly anxiety and depression, in Africa. Further evidence for this claim comes from a meta-analysis of mental health during the COVID-19 pandemic in Africa (Chen et al., 2021), which reported high rates of anxiety, depression, and insomnia. The current study was undertaken in South Africa, where emerging evidence has highlighted a potential mental health crisis that includes unprecedented levels of loneliness, hopelessness, depression, and anxiety (Haag et al., 2022; Pretorius & Padmanabhanunni, 2021b). In South Africa, there is significant inequity in access to resources, and socioeconomic deprivation is prevalent in the form of food insecurity, unemployment, poverty, and low household income, all of which are associated with adverse outcomes (Haag et al., 2022). Studies conducted during the pandemic have highlighted the role of perceived risk of infection and pre-existing mental and physical health conditions in enhancing the risk of COVID-19-related psychological distress (Kim et al., 2022; Pretorius & Padmanabhanunni, 2021b).

Despite the societal increase in mental health disorders during the initial stages of the COVID-19 pandemic, many individuals have been able to effectively cope with new mental health challenges. In addition, many of those who initially suffered psychological distress have returned to pre-pandemic levels of functioning. This trend is evident in several meta-analyses of longitudinal cohort studies comparing mental health before and during the pandemic (Pierce et al., 2021; Robinson et al., 2022). These studies reported that although levels of distress were higher at the outset of the COVID-19 pandemic, these levels either reduced or were comparable to pre-pandemic levels by late 2021. Heightened levels of distress have been related to uncertainty regarding the course of the pandemic, the absence of vaccines at the time, fear of contagion, perceived vulnerability to infection, and the restrictions imposed by the lockdown measures in most countries. Nevertheless, the heterogeneity in responses to adversity underscores the influence of protective factors in promoting coping behaviour. In the context of the pandemic, a range of factors have been identified as protective of mental health, including regular physical activity, social support and maintenance of social contacts, self-efficacy, problem-solving approach, and trust in government policies (Bendau et al., 2021). The current study investigates fortitude as a potential protective resource. Specifically, the study examines the potential direct, mediating, and moderating roles of fortitude in the relationship between perceived vulnerability to disease, fear of COVID-19, and indices of psychological well-being.

Fortitude is defined as the psychological strength to manage adversity and stay well (Pretorius & Padmanabhanunni, 2021a). This strength is gained through adaptive cognitive appraisals of self, family, and supportive others. A range of studies have confirmed the role of fortitude as a protective resource in mental health outcomes among various groups. For example, Padmanabhanunni and Wiid (2022) reported in a study of university students that more fortigenic appraisals were associated with fewer PTSD symptoms. Similarly, Saban (2021) found that fortitude was a significant mediator of mental health outcomes among students with disabilities. Studies among adolescents living in disadvantaged contexts (Pretorius et al., 2016) and nurses caring for patients with Alzheimer’s disease (Heyns et al., 2003) have further confirmed the salient role of fortitude as a protective resource that promotes psychological well-being.

This study is framed within the seminal theory of fortigenesis (Pretorius & Heyns, 1998), which draws from the work of Antonovsky (1996) as well as Lazarus and Folkman (1984; for an overview, see Pretorius & Padmanabhanunni, 2021a). According to this theory, the individual’s subjective cognitive appraisals of stressors are central in influencing their levels of distress and coping. Negative appraisals of one’s capacity to cope with adversity can increase anxiety and lead to adverse outcomes. Similarly, appraising stressors as challenges that can be overcome and perceiving significant others as support resources that can be accessed during times of need can facilitate adaptation (Pretorius & Padmanabhanunni, 2021b). We examined the following hypotheses related to the potential roles that fortitude could play.

Health-sustaining hypotheses:

Hypothesis 1 (H1): High levels of fortitude are associated with low levels of loneliness.
Hypothesis 2 (H2): High levels of fortitude are associated with low levels of anxiety.
Hypothesis 3 (H3): High levels of fortitude are associated with low levels of hopelessness.
Hypothesis 4 (H4): High levels of fortitude are associated with low levels of depression.

Mediating/moderating hypotheses:

Hypothesis 5 (H5): Fortitude mediates/moderates the relationship between germ aversion and loneliness.
Hypothesis 6 (H6): Fortitude mediates/moderates the relationship between germ aversion and anxiety.
Hypothesis 7 (H7): Fortitude mediates/moderates the relationship between germ aversion and hopelessness.
Hypothesis 8 (H8): Fortitude mediates/moderates the relationship between germ aversion and depression.
Hypothesis 9 (H9): Fortitude mediates/moderates the relationship between perceived infectability and loneliness.
Hypothesis 10 (H10): Fortitude mediates/moderates the relationship between perceived infectability and anxiety.
Hypothesis 11 (H11): Fortitude mediates/moderates the relationship between perceived infectability and hopelessness.
Hypothesis 12 (H12): Fortitude mediates/moderates the relationship between perceived infectability and depression.
Hypothesis 13 (H13): Fortitude mediates/moderates the relationship between fear of COVID-19 and loneliness.
Hypothesis 14 (H14): Fortitude mediates/moderates the relationship between fear of COVID-19 and anxiety.
Hypothesis 15 (H15): Fortitude mediates/moderates the relationship between fear of COVID-19 and hopelessness.
Hypothesis 16 (H16): Fortitude mediates/moderates the relationship between fear of COVID-19 and depression.

## Method

### Participants

Participants consisted of a convenience sample of schoolteachers (*n* = 355). Data collection occurred from April to July 2021, which coincided with the period in which the Delta variant of COVID-19 was the dominant strain in South Africa (National Institute for Communicable Diseases, 2021). In response to the rapid rise of infections because of the Delta variant, the South African government raised the national alert (and associated lockdown measures) to Level 4, the second highest level (South African Government, 2022). We were, therefore, unable to implement random sampling, especially because protection of personal information legislation made it challenging to access national databases of teachers. The sample consisted mostly of women (76.6%) who taught at primary school level (61.1%). The mean age of the sample was 41.89 years (*SD* = 12.42, range: 23–73), and the participants’ mean number of years in the teaching profession was 15.7 (*SD* = 11.75, range: 1–48).

### Instruments

Participants completed a demographic questionnaire and the following scales: the Perceived Vulnerability to Disease Questionnaire (PVD-Q: Duncan et al., 2009), the Fear of COVID-19 Scale (FCV-19S; Ahorsu et al., 2020), the Fortitude Questionnaire (FORQ; Pretorius & Heyns, 1998), the UCLA Loneliness Scale (UCLA-LS; Russell, 1996), the trait scale of the State-Trait Anxiety Inventory (STAI-T; Spielberger, 1988), the Beck Hopelessness Scale (BHS; Beck et al., 1974), and the Center for Epidemiological Studies–Depression Scale (CES-D; Radloff, 1977).

The PVD-Q is a 15-item measure that was designed to assess concerns about the transmission of infectious diseases. It consists of two distinct subscales, one of which measures emotional discomfort in situations with a high possibility of disease transmission (Germ Aversion [GA]; eight items), and the other measures beliefs about one’s personal vulnerability to infection (Perceived Infectability [PI]; seven items). Sample items of the GA and PI subscales are: ‘I prefer to wash my hands pretty soon after shaking someone’s hand’ and ‘I am more likely than the people around me to catch an infectious disease’, respectively. Responses to the items are made on a 7-point scale ranging from *strongly disagree* to *strongly agree*. Duncan and colleagues (2009) provided evidence for the convergent, discriminant, and predictive validity of the two subscales, as well as estimates of internal consistency (GA: α = .74, PI: α = .87). Recent studies have reported similar reliability coefficients, with the GA subscale consistently demonstrating lower reliability than the PI subscale (e.g., De Coninck et al., 2020: GA = .70, PI = .85; Tare & Bendre, 2022: GA = .72; PI = .89).

The FCV-19S is a seven-item measure of fear of COVID-19. Participants respond to the items using a 5-point scale ranging from *strongly disagree* to *strongly agree*. An example of an item from the FVC-19S is: ‘I cannot sleep because I’m worrying about getting coronavirus-19’. Ahorsu and colleagues (2020) provided evidence of concurrent validity and reported an alpha coefficient of .82 for the scale. Other studies have reported similar estimates of internal consistency (e.g., Bitan et al., 2020: α = .86; Alyami et al., 2021: α = .88). In South Africa, Pretorius and colleagues (2021) used classical test theory and item response theory to confirm the validity and dimensionality of the FVC-19S and reported satisfactory indices of reliability (α = .91, composite reliability = .91, Mokken scale reliability = .92).

The FORQ measures one’s psychological strength to manage stress and stay well. It consists of 20 items that measure cognitive appraisal in three domains: self-appraisals, family appraisals, and social support appraisals. Responses to the items are made on a 4-point scale ranging from *does not apply* to *applies very strongly*. An example item of the FORQ is: ‘I take a positive attitude towards myself’. Pretorius and Heyns (1998) provided evidence for the validity of the FORQ and reported a reliability coefficient of .85. Other studies have reported similarly satisfactory reliability estimates (e.g., Padmanabhanunni, 2020: α = .89; Yuwanto & Atmadji, 2017: α = .90).

The UCLA-LS is a measure of general loneliness and consists of 20 items. Responses to the items are made on a 4-point Likert-type scale that ranges from *often* to *never.* An example item of the UCLA-LS is, ‘How often do you feel part of a group of friends?’ Russell (1996) provided evidence of convergent and construct validity and reported reliability coefficients that ranged from .89 to .94. Other studies have also reported satisfactory reliabilities (e.g., Shojaee et al., 2021: α = .83; Suri & Garg, 2020: α = .86). Pretorius (2022) confirmed the factor structure of the UCLA-LS and reported reliability coefficients of .92 (α and ω) when used with a sample of students in South Africa.

The STAI-T consists of 20 items and is a measure of trait anxiety. Responses are scored on a 4-point scale that ranges from *almost never* to *almost always*. An example item of the STAI-T is, ‘I am inclined to take things hard’. Spielberger (1988) reported internal consistency estimates of .84 for men and .76 for women. A reliability generalization study reported mean reliability coefficients of .89 and median coefficients of .90, with coefficients ranging from .72 to .96 (Barnes et al., 2002). Pretorius and Padmanabhanunni (2021b) reported a reliability coefficient of .90 for the STAI-T when used with a sample of South African students.

The BHS is a 20-item measure of an individual’s negative expectancies, and participants respond to the items with ‘yes’ or ‘no’. An example item of the BHS is, ‘I just can’t get the breaks’. Beck and colleagues (1974) reported a reliability coefficient (KR-20) of .93, and the association between the BHS and clinical ratings of hopelessness provided evidence for the validity of the scale. More recent studies have reported satisfactory reliability (e.g., Sueki, 2022: KR-20 = .90; Rueda-Jaimes et al., 2018: KR-20 = .90). Padmanabhanunni and Pretorius (2022) reported a KR-20 coefficient of .90 for a sample of South African students.

The CES-D is a 20-item measure of depression that is scored on a 4-point scale ranging from *rarely or none of the time* to *most or all of the time*. An example item of the CES-D is, ‘I thought my life had been a failure’. The author of the scale reported reliability coefficients ranging from .85 to .90. The association between the CES-D and clinical ratings of depression provided evidence for the validity of the scale. More recent applications of the CES-D have reported similarly satisfactory reliability coefficients (e.g., Moeini et al., 2019: α = .85; Faisal et al., 2022: α and ω = .87). Padmanabhanunni and Pretorius (2022) reported a reliability coefficient of .92 for a sample of South African students.

### Procedure

We created an electronic survey of all the measuring instruments using Google Forms. We requested permission from the administrators of Facebook groups consisting of teachers to share the links on their pages. In addition, the school liaison officers of the University of the Western Cape distributed the link to their networks.

### Ethical considerations

The study was conducted in accordance with the guidelines of the Declaration of Helsinki. The research ethics committee at the University of the Western Cape provided ethical clearance (ethics reference number HS21/3/8; 14 May 2021). Participants were assured that participation was voluntary and that their participation was anonymous. Participants had to provide informed consent on the landing page of the link. No personal information was collected and participant confidentiality was maintained.

### Data analysis

**Figure 1.**
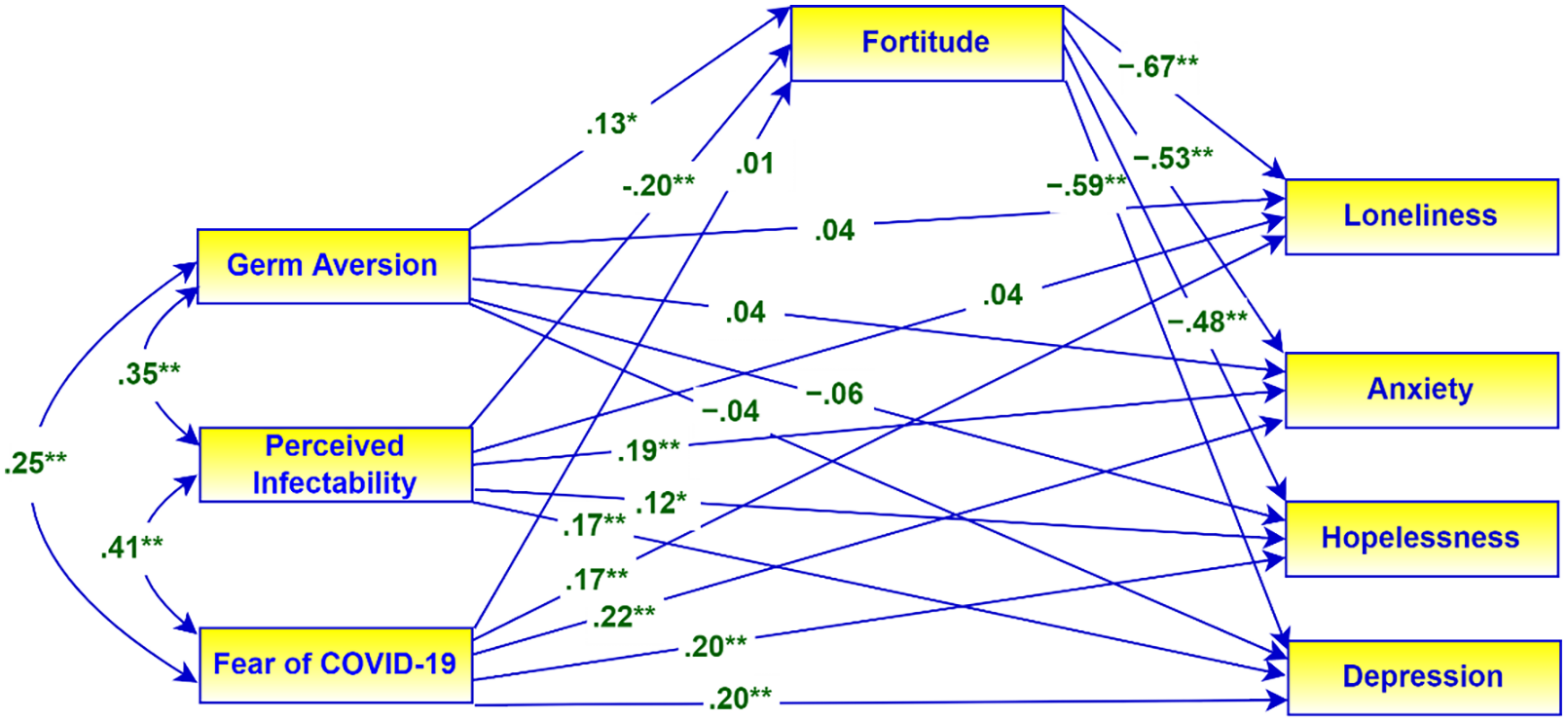
Path analysis model of the mediating role of fortitude. Regression weights are standardized. *p < .05, **p < .001.

IBM SPSS Statistics for Windows (version 28: IBM Corp., Armonk, NY, USA) was used to obtain descriptive statistics (means and standard deviations), intercorrelations between variables (determined using the Pearson’s correlation), and scale reliability (Cronbach’s α and McDonald’s ω). Omega was obtained using the OMEGA macro developed by Hayes and Coutts (2020). To examine the potential mediating role of fortitude, we used path analysis with IBM SPSS Amos (version 26; IBM Corp.). In this regard, maximum likelihood estimations with bootstrapped 95% confidence intervals (CIs) were used to assess the significance of the direct and indirect effects of perceived vulnerability to disease and fear of COVID-19 on indices of psychological distress (see Figure 1). Where no mediation was observed, the potential moderating role of fortitude was examined using the PROCESS macro (Hayes, 2017) in SPSS. In this moderation analysis, the variables used to create the interaction term were mean centered prior to the analysis. The nature of the interaction effect was plotted using the visualization code provided by the PROCESS macro, and the three fortitude subgroups were created using the following option available in the PROCESS macro: −1 *SD*, mean, +1 *SD*.

## Results

The reliabilities of scales, intercorrelations between variables, and descriptive statistics are reported in Table 1. Except for the GA subscale, all other scales demonstrated satisfactory reliability (α and ω: .72–.92). The reliability of the GA subscale (α = .65, ω = .66) can be considered moderate.

**Table 1.** Descriptive statistics, reliabilities, and intercorrelations of study variables.

	1	2	3	4	5	6	7	8
1. Germ aversion	–							
2. Perceived infectability	.35***	–						
3. Fear of COVID-19	.25***	−.41***	–					
4. Fortitude	.06	−.15**	−.04	–				
5. Depression	.04	.33***	.23***	−.63***	–			
6. Anxiety	.13*	.38***	.33***	−.57***	.72***	–		
7. Loneliness	.05	.22***	.21***	−.68***	.65***	.65***	–	
8. Hopelessness	.01	.25***	.25***	−.51***	.61***	.62***	.55***	–
Mean	42.9	28.7	20.9	56.5	22.0	45.0	47.2	5.7
*SD*	8.4	8.8	7.1	11.1	12.2	10.3	11.3	4.9
α	.65	.78	.91	.92	.92	.91	.92	.89
ω	.66	.78	.91	.92	.92	.91	.92	.89

*SD*: standard deviation.

*** *p* < .001, **p < .01, *p < .05.

The findings in Table 1 indicate that germ aversion was positively related to anxiety (*r* = .13, *p* = .02), which means that high levels of germ aversion were associated with high levels of anxiety. Perceived infectability was positively related to all indices of psychological well-being: depression (*r* = .33, *p* < .001), anxiety (*r* = .38, *p* < .001), loneliness (*r* = .22, *p* < .001), and hopelessness (*r* = .25, *p* < .001). Thus, high levels of perceived infectability were associated with high levels of depression, anxiety, loneliness, and hopelessness. Fortitude was negatively related to all indices of psychological well-being: depression (*r* = −.63, *p* < .001), anxiety (*r* = −.57, *p* < .001), loneliness (*r* = −.68, *p* < .001), and hopelessness (*r* = −.51, *p* < .001). Thus, high levels of fortitude were associated with low levels of depression, anxiety, loneliness, and hopelessness.

The path analysis model used to examine the mediating effects of fortitude is presented in Figure 1 with the associated standardized regression coefficients. In this model, perceived vulnerability to disease (GA and PI) and fear of COVID-19 are the predictors, and the indices of psychological well-being are the dependent variables. Fortitude is the presumed mediator.

The results of the mediation analysis reflected in Figure 1 are presented in Table 2. The results in Table 2 provide support for the following hypotheses:

**Table 2.** Direct and mediating effects of fortitude on indices of psychological well-being.

Effect	*B*	*SE*	β	95% CI	*p*
Direct effects					
Fortitude → depression	−.65	.04	−.59	[−.65, −.52]	.001
Fortitude → hopelessness	−.21	.02	−.48	[−.55, −.41]	.001
Fortitude → anxiety	−.50	.04	−.53	[−.59, −.47]	.001
Fortitude → loneliness	−.68	.04	−.67	[−.72, −.62]	.001
Mediating effects					
PI → fortitude → depression	.16	.05	.12	[.08, .25]	.001
PI → fortitude → hopelessness	.05	.02	.10	[.03, .08]	.001
PI → fortitude → anxiety	.12	.04	.11	[.06, .19]	.001
PI → fortitude → loneliness	.17	.05	.13	[.09, .26]	.001
GA → fortitude → depression	−.11	.05	−.08	[−.19, −.03]	.014
GA → fortitude → hopelessness	−.04	.02	−.06	[−.06, −.01]	.013
GA → fortitude → anxiety	−.08	.04	−.07	[−.14, −.03]	.015
GA → fortitude → loneliness	−.12	.05	−.09	[−.20, −.04]	.014
FCV → fortitude → depression	−.01	.06	−.01	[−.12, −.09]	.841
FCV → fortitude → hopelessness	−.01	.02	−.01	[−.04, .03]	.845
FCV → fortitude → anxiety	−.01	.05	−.01	[−.09, .07]	.843
FCV → fortitude → loneliness	−.01	.07	−.01	[−.12, .10]	.839

*SE*: standard error; CI: confidence interval; PI: perceived infectability; GA: germ aversion; FCV: fear of COVID-19.

H1–H4: High levels of fortitude were associated with low levels of loneliness (β = −.67, 95% CI = [−.27, −.62], *p* = .001), anxiety (β = −.53, 95% CI = [−.59, −.47], *p* = .001), hopelessness (β = −.48, 95% CI = [−.55, −.41], *p* = .001), and depression (β = −.59, 95% CI = [−.65, −.52], *p* = .001).
H5–H8: Fortitude partially mediated the relationship between germ aversion, on one hand, and loneliness (β = −.09, 95% CI = [−.20, −.04], *p* = .014), anxiety (β = −.07, 95% CI = [−.14, −.03], *p* = .015), hopelessness (β = −.06, 95% CI = [−.06, −.01], *p* = .013), and depression (β = −.08, 95% CI = [−.19, −.03], *p* = .014), on the other hand.
H9: Fortitude fully mediated the relationship between perceived infectability and loneliness (β = .13, 95% CI = [.09, .26], *p* = .001). In the absence of the mediator, loneliness was significantly associated with perceived infectability (β = .22, 95% CI = [.14, .33], *p* = .001), but this association was non-significant in the presence of the mediator.
H10–H12: Fortitude partially mediated the relationship between perceived infectability, on one hand, and anxiety (β = .11, 95% CI = [.06, .19], *p* = .001), hopelessness (β = .10, 95% CI = [.03, .08], *p* = .001), and depression (β = .12, 95% CI = [.08, .25], *p* = .001), on the other hand.

However, fortitude did not mediate the relationship between fear of COVID-19, on one hand, and loneliness (β = −.01, 95% CI = [−.12, 10], *p* = .839), anxiety (β = −.01, 95% CI = [−.09, 07], *p* = .843), hopelessness (β = −.01, 95% CI = [−.04, 04], *p* = .845), and depression (β = −.01, 95% CI = [−.12, 09], *p* = .841), on the other hand. Therefore, we examined the potential moderating role of fortitude in the relationship between fear of COVID-19 and indices of psychological well-being. The results of the moderation analysis are presented in Table 3.

**Table 3.** Moderating role of fortitude in the relationship between fear of COVID-19 and indices of psychological well-being (product term is fear of COVID-19 × Fortitude).

Dependent variable	*B*	*SE*	*t* value	95% CI	*p*
Depression	.013	.005	2.36	[.002, .024]	.02
Loneliness	−.002	.005	−0.33	[−.011, .008]	.74
Anxiety	.005	.004	1.16	[−.004, .015]	.25
Hopelessness	.000	.002	−0.05	[−.005, .005]	.96

*SE*: standard error; CI: confidence interval.

**Figure 2.**
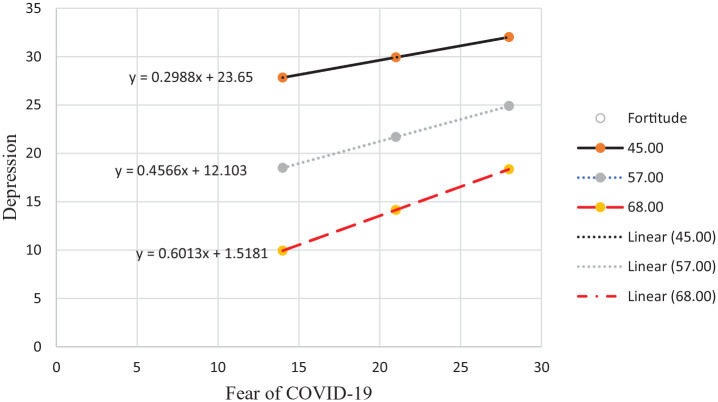
Moderating role of fortitude in the relationship between fear of COVID-19 and depression. 45 = low fortitude (solid line), 57 = moderate fortitude (dotted line), 68 = high fortitude (dashed line).

The results in Table 3 indicate that fortitude moderated the relationship between fear of COVID-19 and depression (*B* = .013, 95% CI = [.002, .024], *p* = .02) but did not moderate the relationships between fear of COVID-19, on one hand, and loneliness (*B* = −.002, 95% CI = [−.011, .008], *p* = .74), anxiety (*B* = .005, 95% CI = [−.004, .015], *p* = .25), and hopelessness (*B* = .000, 95% CI = [−.005, .005], *p* = .96), on the other hand. The nature of the interaction between fear of COVID-19 and fortitude is plotted in Figure 2.

Figure 2 indicates that at both low and high levels of fear of COVID-19, participants with low and moderate fortitude reported higher levels of depression than participants with high fortitude.

## Discussion

The COVID-19 pandemic has had a disruptive impact on many aspects of life. The pandemic and its containment measures (e.g., national lockdowns, social distancing protocols, and work-from-home mandates) precipitated distress and adversely impacted psychological health and well-being for many people (Robinson et al., 2022). The mental health impact of COVID-19 has been well documented in recent literature, yet comparatively few studies have investigated the role of protective factors that facilitate coping. Emerging evidence indicates that despite the distress initially evoked by the pandemic, a significant portion of the population across various countries have been able to effectively adapt, and many people have returned to pre-pandemic levels of functioning (O’Connor et al., 2021; Pierce et al., 2021). This evidence suggests that protective factors are at play in counterbalancing the potentially damaging effects of the pandemic. Further investigation is warranted to identify these sources of strength. The current study examined the potential direct, mediating, and moderating roles of fortitude in the relationship between perceived vulnerability to disease, fear of COVID-19, and indices of psychological well-being. There were several significant findings.

First, fortitude was found to have a health-sustaining effect, evident in its association with all indices of psychological well-being. This finding lends support to the hypothesis of fortitude as a protective resource. Although existing research on the construct of fortitude is limited in comparison to studies of other protective factors (e.g., social support, resilience), it is possible to contrast the current study findings with those of studies of the role of self-efficacy in coping with adversity. The construct of general self-efficacy, which is defined as a stable sense of confidence in one’s ability to manage difficulty (Zeng et al., 2021), is similar to fortigenic appraisals of self. General self-efficacy influences the way threats are perceived, and studies conducted during the pandemic have provided evidence that high levels of self-efficacy are associated with better coping and reduced distress. For example, a study of stress and quality of life among intensive care nurses during the pandemic (Peñacoba et al., 2021) reported that greater perceptions of self-efficacy were associated with lower perceptions of stress and higher levels of resilience. Similarly, Zeng et al. (2021) found that college students who had high general self-efficacy (i.e., strong beliefs about their capacity to cope and problem solve) were better able to deal with pandemic-related adversity than peers with lower self-efficacy. Furthermore, self-efficacy has been found to be a salient predictor of health-promoting behaviours. In a study by Shahnazi et al. (2020), participants with higher perceived self-efficacy were more inclined to adopt preventive behaviours in relation to the COVID-19 pandemic than those with lower perceived self-efficacy. An individual’s appraisals that they are doing what is needed to safeguard themselves and their families can contribute to feeling protected, which in turn can promote well-being. Individuals with higher self-efficacy are also more inclined than others to use solution-focused coping strategies (González-Castro et al., 2021). Applied to the construct of fortitude, these findings suggest that those with greater positive appraisals of their own capacity to manage adversity may be more inclined to engage in adaptive coping responses than their peers. The use of solution-focused coping strategies has been associated with improved well-being and mental health (González-Castro et al., 2021).

Second, fortitude mediated the relationship between perceived vulnerability to disease (i.e., germ aversion and perceived infectability) on one hand, and loneliness, depression, anxiety, and hopelessness on the other. The existing studies have confirmed that high perceived risk of infection is associated with a range of adverse mental health outcomes. Appraising oneself as being more vulnerable to infection than others is likely to heighten fear of COVID-19 and anxiety related to the impact of contracting the virus (Yıldırım et al., 2022). High perceived vulnerability to infection can heighten an individual’s sensitivity to cues that signal disease (e.g., someone coughing or sneezing), resulting in interpersonal avoidance and social isolation. In turn, the absence of social connection can lead to loneliness and depression (Boyraz et al., 2020). Based on the results of the current study, it is probable that those with higher fortigenic appraisals of self and others are more able than others to construe their worries about risk of infection as rational and adaptive and perceive their engagement in preventive measures as a means of safeguarding their well-being and that of significant others. These appraisals may serve to counterbalance adverse mental health outcomes.

Third, fortitude moderated the relationship between fear of COVID-19 and depression. At both high and low levels of fear of COVID-19, those with low and moderate levels of fortitude reported higher levels of depression than those with higher levels of fortitude. Similar results have been reported in studies of the construct of resilience. For example, Yıldırım et al. (2022) found that resilience mediated the relationship between fear of COVID-19 and depression, anxiety, and stress among health care professionals. Fortitude is grounded in a theory of cognitive appraisal, whereas resilience is conceptualized as the capacity to adapt to challenging situations. It can nevertheless be argued that fortitude (i.e., positive appraisals of self, family, and significant others) enables one to be resilient when confronted with adversity. In the current study, it is probable that appraising fear of COVID-19 as reasonable and viewing others as a resource that can be drawn upon to manage the stressors associated with the pandemic mitigated the development of severe distress. Furthermore, those with high fortigenic self-appraisals are able to draw on active problem-solving skills to address challenges, which may preserve mental health (Pretorius & Padmanabhanunni, 2021a) and account for the current study findings.

These findings have theoretical and practical implications. On a theoretical level, the study further extends support for the role of fortitude as a protective resource in the context of a public health emergency. The significant moderating and mediating effects of fortitude on psychological outcomes support its potential for counterbalancing the negative impacts of the COVID-19 pandemic on mental health. From an intervention perspective, enhancing fortitude among vulnerable population groups can serve to enhance their capacity to cope with adversity. Fortitude is based on a theory of appraisal and, according to social-cognitive theory, persistent negative appraisals generated in relation to stressful or traumatic events underlie depression, anxiety, and loneliness and lower well-being. Fortigenic appraisals of self and others facilitate coping through a reappraisal process. People who have more positive fortigenic appraisals are better able to reappraise stressors as challenges and believe that they have the internal capacities and external supports and resources to overcome adversity (Pretorius & Padmanabhanunni, 2021a). Hence, enhancing fortigenic appraisals can potentially facilitate coping.

Cognitive-behavioural therapy (CBT) interventions focus on targeting problematic negative cognitive appraisals and maladaptive thinking patterns that underlie psychopathology and such interventions may hold promise in enhancing fortitude. Acceptance and commitment therapy is a form of CBT that focuses on perspective-taking and promotion of psychological flexibility (Chong et al., 2021), and this approach could be adapted to reinforce fortigenic appraisals and build psychological strength. In addition to in-person sessions, alternative modes of delivering these interventions, such as via smartphone applications and video conferencing technology, could reach a wider audience and help to protect mental health during public health emergencies (Chong et al., 2021).

The study has certain limitations. First, the participants sampled were mostly women from a single demographic group (i.e., school teachers) and mostly resided in a distinct geographic area. Future studies using more diverse samples would be beneficial to corroborate the results. Second, a cross-sectional survey research design was used, which limits the ability to draw causal inferences. The results reflect the psychological status of the sample at a specific time point, and longitudinal studies are necessary to explore long-term interactions between the study variables. A third limitation of the study is the reliance on electronic self-report instruments. This method is susceptible to social desirability bias and common method variance, which can result in an exaggeration of the study associations. A multi-method approach may be beneficial to enhance the rigour of future studies.

## Conclusion

The study provides support for the role of fortitude as a health-sustaining resource in the context of a public health emergency. The significant moderating and mediating effects of fortitude on psychological outcomes support its potential for counterbalancing the negative impacts of the COVID-19 pandemic on mental health. Interventions aimed at enhancing fortigenic appraisals of self and others may prove beneficial in promoting psychological well-being.
